# The Principles of Gynæcology

**Published:** 1912-12

**Authors:** 


**The Principles of Gynæcology.** By W. Blair Bell, B.S., M.D. Pp. xxvii, 551. London: Longmans, Green & Co. 1910. Price 21s. net.

We have read this book with much enjoyment and profit. It is written in a clear and pleasant style, and the orderly and scientific arrangement of the subject-matter in chapters and sections, each of which stimulates interest in its successor, admirably helps to sustain the reader's interest.

Beginning with an account of the evolution of the female genital organs the author passes on to describe their development in the human female, and shows how the evolutionary history of the race is recapitulated in the development of the individual, and how a knowledge of evolution and development is an aid to the understanding of normal anatomy and of malformations. The next portion of the book deals with the normal anatomy and physiology of the parts, and then with congenital and acquired derangements of these normal conditions. Following this come a chapter on infective and parasitic diseases of the genital tract, three chapters on tumours—cysts, innocent growths, and malignant growths—and, lastly, a short account of general diseases or diseases of neighbouring parts in so far as they affect the pelvic organs. The book concludes with two good chapters on gynæcological surgery.

In two appendices are found a consideration of the subject of electro-therapeutics in relation to diseases of women and a classification of the causes of some of the most prominent symptoms met with in gynæcological work.

It will thus be seen that the usual anatomical arrangement of the subject has been somewhat widely departed from, and a more pathological method of compilation adopted. In the main this is good, especially in the case of infective diseases, the proper understanding of which is much facilitated by considering them as affections of the genital tract as a whole with a possible special incidence on some particular segment. So, too, in the matter of cysts and new growths it is of great assistance in obtaining a grasp of the subject to consider the reproductive organs as a whole, as for instance in the case of the various cysts which may arise from Wolffian relics. But while we think that the author's arrangement immensely increases the interest in reading the book, and is of the utmost value in enabling the reader to get a clear and thorough understanding of the subject, yet we are of opinion that a chapter summarising the conditions which may affect individual organs would be a help to the student in answering examination questions and also to the practitioner in diagnosis. For instance, there may be in a given patient a swelling which is found on examination to be connected with the tube, and in such a case it is helpful to be able to run rapidly over in the mind the various conditions which may produce enlargement of the tube. This is more easily done by the student if his text-book has been written on the anatomical basis, but the need might be equally met by means of a third appendix.

A feature of the book is the number and excellence of the illustrations. The microphotographs are particularly good, and are much to be preferred to drawings of sections made by hand, which always tend to be too diagrammatic. Another noteworthy point is the discussion of the indications for treatment in different conditions, for instance in uterine displacements. The different effects of the condition under consideration in different classes of patients are shown and plain rules for the practitioner's guidance deduced.

In his preface the author states that he has not attempted to give alternative views on disputed points, but has given the view which most commends itself to him. This makes very greatly for clearness, of course, and is therefore good, but there are certain cases in which we think alternative views should have been each clearly put, and the reasons for the choice of one given. One such case is that of "endometritis." There is nothing in gynæcology more confusing to the student than the conditions included under this designation. The author describes acute septic endometritis and gonorrhœal endometritis in their appropriate sections; senile endometritis is very briefly referred to elsewhere; and the so-called "chronic endometritis" is dealt with under the heading of diffuse adenoma of the endometrium. In all this we are inclined to agree, but we think that, seeing that some authorities still use the term "chronic endometritis" for this adenomatous condition, and how very confusing the subject is to the student, it would have been well to gather up into one place all the conditions known as endometritis, to classify them and mention their points of difference, and show why some are really inflammatory and deserving of the name endometritis while others are not. This might be done, to avoid introducing ambiguities in the text, by means of a somewhat extended footnote.

The subject of chorion-epithelioma merits a somewhat longer account than is given to it in this book. Apart from the great interest of the condition, its pathology is obscure enough, as the history of its recognition shows, to demand a somewhat detailed account, if it is to be understood by one just commencing the study of gynæcology.

The descriptions of the operations of gynæcological surgery at the end of the book, only those operations being described which commend themselves to the author himself, are easy to follow, and the illustrations and explanatory diagrams are clear and helpful.

This book, in short, shows distinctive features enough to justify its existence, and seems to us to present the subject in a concise, scientific, and withal most interesting manner. For its scientific basis and the excellence of the pathological descriptions and plates we can heartily recommend it to the student, while the definitely expressed and reasoned recommendation of the author with regard to treatment cannot fail to be of great assistance to practitioners.

